# Catch-up growth in children with chronic kidney disease started on enteral feeding after 2 years of age

**DOI:** 10.1007/s00467-019-04382-9

**Published:** 2019-10-24

**Authors:** Matko Marlais, Jelena Stojanovic, Helen Jones, Shelley Cleghorn, Lesley Rees

**Affiliations:** 1 Great Ormond Street Hospital for Children NHS Foundation Trust, Great Ormond Street, London, WC1N 3JH UK; 2 UCL Great Ormond Street Institute of Child Health, London, UK; 3 Evelina London Children’s Hospital, London, UK

**Keywords:** Paediatrics, Chronic kidney disease, Growth, Enteral feeding, Gastrostomy

## Abstract

**Background:**

Enteral feeding by tube in chronic kidney disease (CKD) before 2 years of age improves growth. Whether it is effective after this age is unknown. We assessed whether height and weight SDS changed after tube feeding was started in children with CKD above 2 years of age.

**Methods:**

Retrospective study of pre-transplant, pre-pubertal children (< 11 years) with CKD stages 2–5 started on nasogastric tube or gastrostomy feeds for the first time after age 2 years. Children were identified by searching dietetic records and the renal database. Children on growth hormone were excluded. Height, weight, and BMI were documented 1 year prior to and at the start of tube feeds, and after 1 and 2 years. Data collection ceased at transplantation.

**Results:**

Fifty children (25 male) were included. The median (range) age at start of tube feeds was 5.6 (2.1–10.9) years. Sixteen children were dialysed (1 haemodialysis, 15 peritoneal dialysis); 34 predialysis patients had a median (range) eGFR of 22 (6–88) ml/min/1.73 m^2^. Overall height SDS (Ht SDS) improved from − 2.39 to − 2.27 at 1 year and − 2.18 after 2 years (*p* = 0.02). BMI SDS improved from − 0.72 to 0.23 after 1 year and was 0.09 after 2 years of enteral feeding (*p* < 0.0001). Height SDS improved more in children aged 2–6 years (− 2.13 to − 1.68, *p* = 0.03) and in children not on dialysis (− 2.33 to − 1.99, *p* = 0.002).

**Conclusions:**

Enteral tube feeding commenced after 2 years of age in prepubertal children with CKD improves height and weight SDS, with stability of BMI during the second year. Younger children and those not on dialysis had the greatest benefit.

## Introduction

Poor growth is a recognised complication of chronic kidney disease (CKD) across all stages but is especially notable in CKD stage 5, both before and on dialysis [1]. Infants and children less than 2 years of age are particularly at risk as adequate nutrition, which can be difficult to maintain, is the main determinant of growth at this age [2]. Improvement of growth can be achieved with careful attention to the optimisation of nutrition, bone disease, anaemia and salt, water and acid base metabolism [3].

After the infantile phase of growth, disturbances of the growth hormone (GH) IGF1 axis with GH resistance play a bigger role. Renal transplantation induces catch-up growth in younger children with CKD [4, 5]. Recombinant human GH (rhGH) also improves growth [6] and is recommended when nutrition, metabolic control and anaemia have been optimised.

While there is good evidence that enteral tube feeding during the first 2 years of life improves both weight SDS (Wt SDS) and height SDS (Ht SDS) in children with CKD [7–10], the evidence for any benefits over this age is conflicting [2, 11]. Dietary advice improved Ht SDS in 13 children with eGFR < 25 ml/min/1.73 m^2^ who were 2–16 years of age [12]. Other smaller studies have failed to find improvement in growth after nutritional support or tube feeding in children with CKD after the age of 2 years [7, 8, 13, 14].

The primary aim of this study was to assess whether enteral tube feeding started after the age of 2 years was associated with a change in Ht SDS in children with CKD. Secondary aims were to assess whether enteral tube feeding was associated with changes in Wt SDS, body mass index (BMI) SDS, serum albumin and parathyroid hormone (PTH).

## Methods

### Participants

This is a retrospective study of pre-pubertal children with CKD stages 2–5 who started enteral tube feeding after the age of 2 years and before the age of 11 years. Patients were identified by searching dietetic records and the renal database at one large tertiary paediatric nephrology unit (Great Ormond Street Hospital for Children NHS Foundation Trust). Formal ethical approval was not required for this retrospective study, but all data were recorded and stored anonymously, and data protection regulations were adhered to.

Patients were consecutively included if they were followed up for at least 2 years after starting enteral tube feeding and if full healthcare records including anthropometric data were available. All children were included if they met the inclusion criteria during the period 1998–2018. Children who received nasogastric tube feeding limited to the neonatal period but were not tube-fed again until after 2 years of life were still considered for inclusion in this study. Enteral feeding was either through nasogastric or gastrostomy tube. Patients were excluded from this study if they were on rhGH treatment; data collection ceased if the child received a renal transplant.

The practice of the unit is that children are routinely reviewed at each clinic visit by a renal dietician as well as a nephrologist. Dietary intake is assessed, and dietary supplements of energy and/or protein are recommended if height and weight are falling away from centiles, and intake is less than that recommended for the normal population of the same age. Growth continues to be assessed at each clinic visit and the feed prescription adjusted accordingly.

### Data collection

Data were collected on baseline characteristics including age, sex, primary renal diagnosis and significant comorbidities. Type of enteral tube feeding was documented. Children were recorded as on dialysis at the time of starting tube feeds or not on dialysis: for the latter their GFR was estimated according to the Schwartz formula [15]. It was also recorded if a child started dialysis subsequent to starting enteral tube feeding.

Anthropometric data (height and weight) were collected 1 year before starting enteral feeding, at the time of starting, and 1 and 2 years after starting enteral feeding. In children on dialysis, an estimated dry/optimum weight was used to avoid discrepancies due to fluid status. Height was measured by trained clinical staff using a stadiometer as per standard clinical practice. Ht SDS, Wt SDS and BMI SDS were calculated using UK-WHO growth data [16, 17]. Where available, data were collected on serum albumin and serum intact PTH level (normal range 0.7–5.6 pmol/L) at each of the data collection time points.

Due to the retrospective nature of this study we allowed a 2-month window either side of the 1 and 2 year data collection, as not all children would have been routinely seen or had their height and weight documented at those exact time points. The 2-month window was decided upon pragmatically, and SDS were calculated based on the age of the child at the time of the clinical review.

### Statistical analyses

Comparisons of anthropometric data were carried out across the study time points baseline, 1 year and 2 years of enteral tube feeding, using one-way repeated measures. Analysis of variance (ANOVA) after data was checked for normal distribution using the Shapiro-Wilk test. Serum albumin and PTH were analysed at the time of starting enteral tube feeds and 1 and 2 years after this; these were compared using the Wilcoxon signed-rank test as these data were not normally distributed.

Subgroup analyses were undertaken by dividing the study population into those aged 2–6 years and those aged 6–11 years at the start of enteral tube feeding. The study population was also divided into those on dialysis and not on dialysis at the start of tube feeding.

Where data were missing, paired analysis was not undertaken but their data are included in summary statistics. Statistical analyses were performed using SPSS version 22 (SPSS Inc, Chicago, Illinois). All tests were two-tailed and a *p* value < 0.05 was taken to represent a statistically significant result.

## Results

Fifty children were included. Table 1 shows baseline characteristics. Sixtteen children were dialysed (1 haemodialysis (HD), 15 peritoneal dialysis (PD)); 34 had a median (range) eGFR of 22 (6–88) ml/min/1.73 m^2^. Only 4 children had an eGFR > 60 ml/min/1.73 m^2^ (two with cystinosis, one with Bartter syndrome and one with nephrogenic diabetes insipidus). Table 1 also shows the primary renal diagnoses of children included in this study. Ten children had significant comorbidity in association with their primary renal diagnosis (7 children with neurodevelopmental problems, 1 child with haemoglobinopathy, 2 children had multiple congenital abnormalities e.g. CHARGE, VACTERL).

**Table 1:** Baseline characteristics and primary renal diagnosis of 50 pre-pubertal children with CKD started on tube feeding above 2 years of age, including sub-groups

	All children (*n* = 50)	2–6 years (*n* = 29)	6–11 years (*n* = 21)	Dialysis (*n* = 16)	Pre-dialysis (*n* = 34)
Median age in years (range)	5.6 (2.1–10.9)	3.7 (2.1–5.9)	7.5 (6–10.9)	6.7 (2.1–10.9)	4.8 (2.1–10.9)
Number male (%)	25 (50%)	19 (66%)	6 (29%)	6 (38%)	19 (56%)
Primary renal diagnosis					
CAKUT	17 (34%)	15 (52%)	2 (10%)	4 (25%)	13 (38%)
Nephronophthisis	8 (16%)	2 (7%)	6 (29%)	4 (25%)	4 (12%)
FSGS	7 (14%)	3 (10%)	4 (19%)	4 (25%)	3 (9%)
Cystinosis	7 (14%)	3 (10%)	4 (19%)	1 (6%)	6 (18%)
Other tubulopathies or stones	5 (10%)	3 (10%)	2 (10%)	0 (0%)	5 (15%)
Other causes	6 (12%)	3 (10%)	3 (14%)	3 (19%)	3 (9%)

*CAKUT* congenital anomalies of the kidney and urinary tract (including posterior urethral valves, vesico-ureteric reflux and renal dysplasia), *FSGS* focal segmental glomerulosclerosis (including steroid resistant nephrotic syndrome)

Ten children were transplanted after the start of enteral tube feeding and so 2-year follow-up data were not collected for these children. Thirty-nine children were fed through a gastrostomy tube; 6 children were initially fed through a nasogastric tube then converted to a gastrostomy during the study period; 5 children were fed through a nasogastric tube throughout.

**Fig. 1:**
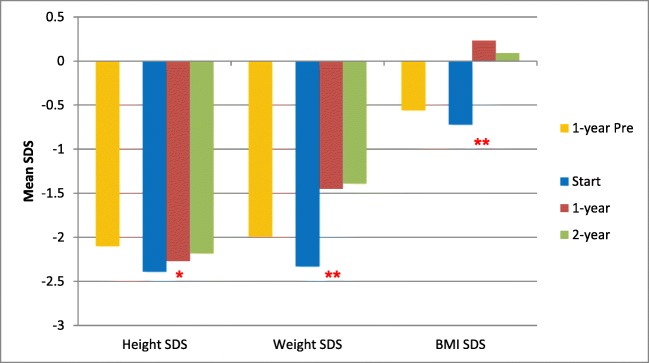
Height, weight and BMI SDS of 50 children 1 year prior to and at the start of enteral tube feeding, and at 1 and 2 year follow up. *p < 0.05 and **p < 0.01 for difference across three time periods (one-way repeated measures ANOVA, three groups compared were at start of enteral tube feeds, 1-, and 2-year follow-up). BMI body mass index, SDS standard deviation score

Table 2 and Fig. 1 show the results for anthropometric data across the study. The mean height velocity across the 2 years of the study was 6.3 cm/year in the 40 children where 2-year follow-up data were available; the mean height velocity SDS was − 0.48. Median (range) serum albumin increased from 41.5 g/L (19–51) at the start of enteral feeding to 42 g/L (33–49) after 2 years (*p* = 0.03, Wilcoxon signed-rank test, data missing for 13 children). Median (range) serum intact PTH decreased from 8.4 pmol/L (0.3–156) at the start to 5.6 pmol/L (0.5–67) after 2 years but this was not statistically significant (*p* = 0.08, Wilcoxon signed-rank test, data missing for 23 children).

**Table 2:** Height, weight and BMI SDS of 50 children 1 year prior to and at the start of enteral tube feeding, and at 1- and 2-year follow-up

	1 year prior to starting tube feeds (*n* = 30)	At start of tube feeds (*n* = 50)	1-year post tube feeds (*n* = 49)	2-year post tube feeds (*n* = 40)	*p* value (one-way repeated measures ANOVA)
Mean (±SD) height SDS	− 2.1 (± 1.42)	− 2.39 (± 1.49)	− 2.27 (± 1.39)	− 2.18 (± 1.51)	0.02
Change in height SDS		0	+ 0.12	+ 0.21	
Mean (±SD) weight SDS	− 1.99 (± 1.65)	− 2.33 (± 1.76)	− 1.45 (± 1.66)	− 1.39 (± 1.86)	< 0.0001
Change in weight SDS		0	+ 0.88	+ 0.94	
Mean (±SD) BMI SDS	− 0.56 (± 1.29)	− 0.72 (± 1.49)	0.23 (± 1.33)	0.09 (± 1.47)	< 0.0001
Change in BMI SDS		0	+ 0.95	+ 0.81	

*BMI* body mass index, *SDS* standard deviation score, *ANOVA* analysis of variance. ANOVA compares only three groups (start of enteral feeds, 1-, and 2- year follow -up)

*BMI* body mass index, *SDS* standard deviation score, *ANOVA* analysis of variance. ANOVA compares only three groups (start of enteral feeds, 1-, and 2-year follow-up)

Data on anthropometry 1 year prior to starting enteral tube feeding was available for 30 children as some children were started on tube feeds soon after presenting with CKD. These data were not analysed statistically but is presented in Table 2 and Fig. 1 to demonstrate the patient’s trajectory prior to starting enteral feeds. The mean height velocity prior to starting tube feeds was 5.6 cm/year with a mean height velocity SDS − 1.51.

**Fig. 2:**
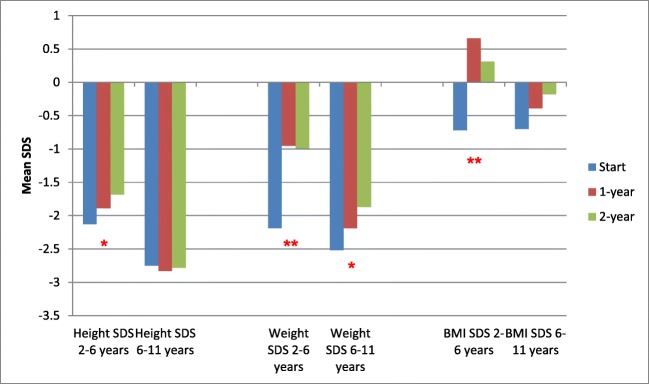
Height, weight and BMI of 50 children at the start of enteral tube feeding and at 1- and 2-year follow-up, subdivided by age of starting tube feeds (29 children aged 2–6 years and 21 children aged 6–11 years). *p < 0.05 and **p < 0.01 for difference across three time periods (one-way repeated measures ANOVA). BMI body mass index, SDS standard deviation score

**Fig. 3:**
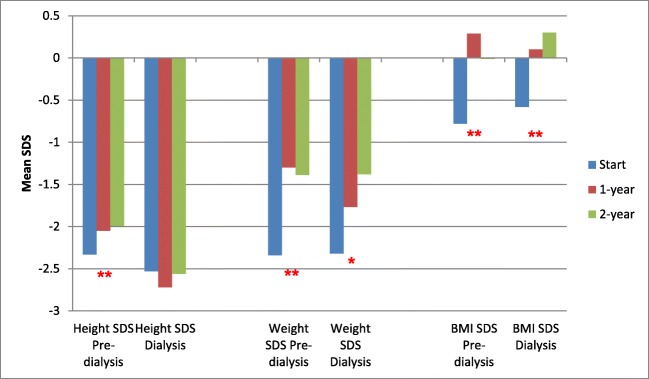
Height, weight and BMI of 50 children at the start of enteral tube feeding and at 1- and 2-year follow-up, subdivided into 16 children on dialysis and 34 children pre-dialysis when starting tube feeds. *p < 0.05 and **p < 0.01 for difference across three time periods (one-way repeated measures ANOVA). BMI body mass index, SDS standard deviation score

Figures 2 and 3 show the subgroup analyses. Figure 2 shows the differing results when the study cohort is split into 29 children aged 2–6 years and 21 children 6–11 years at the time of starting enteral tube feeds. Figure 3 shows the differing results when the cohort is split into 34 children not on dialysis and 16 children on dialysis at the time of starting tube feeds. Improvements in height SDS were seen only in children aged 2–6 years and children not on dialysis at the time of starting tube feeds. Improvements in weight SDS were, however, seen across all ages and in those on and not on dialysis at the time of starting enteral feeding. Of the 34 children not on dialysis at the time of starting tube feeds, 7 were started on dialysis during the 2-year follow-up period of the study (5 on peritoneal dialysis, 2 on haemodialysis) with a median time of starting dialysis 10 months after starting tube feeds (range 3–18 months).

## Discussion

These results demonstrate that enteral tube feeding started after 2 years of age in pre-pubertal children with CKD was associated with an improved Ht and Wt SDS with normalisation of BMI. There is no evidence of excessive weight gain in this cohort, with BMI SDS remaining stable in the second year. Subgroup analyses show that improvements in Ht SDS are limited to those aged 2–6 years of age and those not already on dialysis at the time of starting enteral feeds. To our knowledge, this is the largest study to date assessing the impact of enteral feeding in this older cohort of children with CKD.

Although some previous smaller studies have not demonstrated improved Ht SDS with enteral tube feeding after the age of 2 years, Norman et al. found an improved rate of growth with regular dietary advice [12]. Our findings with regard to improvements in Wt SDS seen across our cohort are in agreement with previously published data [8, 14], but importantly, our study demonstrates through the normalised BMI that excessive weight gain does not seem to occur in children continuing on enteral tube feeds for 2 years. This may be due to the regular dietetic reviews ensuring that energy intake was sufficient to support growth but not excessive.

Interestingly, height velocity SDS was lower than average at − 0.48 across the 2-year study period. We cannot assess the relative impact of enteral feeding on height velocity as we did not have a comparator group, and it may be that height velocity would be even lower than this had enteral tube feeds not been started. Indeed, this is suggested as the height velocity SDS prior to starting tube feeds was − 1.51 in those children where data were available. However, this finding does suggest that children with CKD have lower than average height velocity even after improvement of their nutritional state. This group can then be reassessed for rhGH therapy.

It may seem counterintuitive that height SDS increased overall, despite a negative height velocity SDS in the study. This has occurred as the height deficit of children at the start of the study was of a greater magnitude than the deficit in height velocity; therefore, to maintain the height deficit, an equivalent deficit in height velocity would be needed. In this study, the height improved overall as the height velocity was only slightly less than normal during the study (SDS − 0.48), but the original height was significantly less than normal (SDS − 2.39).

Our study also found that serum albumin increased significantly after starting enteral tube feeds. This may be a reflection of the improved nutritional state as demonstrated by anthropometric data, but it is acknowledged that the use of albumin as a marker of nutritional state is controversial in CKD [18]. We also acknowledge that although the increase in albumin levels is statistically significant, it is not clinically significant.

Although data on height and weight 1 year prior to starting feeds were only available for 30 children, these data suggest that Ht and Wt SDS were in fact deteriorating in this cohort of children prior to the commencement of enteral feeds. It would appear this downward trend was halted by the commencement of enteral tube feeds as Ht and Wt SDS improved after starting tube feeds.

It is interesting to note that the improvement in Wt SDS and normalisation of BMI SDS occurred almost entirely in the first year of enteral tube feeding, with the second year showing maintenance of similar values without significant additional improvement. Ht SDS, however, did show progressive improvement across the two years. This suggests that, although nutritional state can be optimised quite quickly after starting enteral tube feeds, the maintenance of tube feeding may still assist with improvements in height.

Subgroup analyses have revealed that in our cohort, only those aged 2–6 years had a significant improvement in their Ht SDS; the older children aged 6–11 years did not. This may be due to a lack of power with the reduced numbers in each subgroup, but it may also be a true finding reflecting the continuum of influences on growth, with nutrition being a major factor in younger children (but not limited to those aged less than 2 years). Our study also shows that significant improvements in Ht SDS were limited to children not yet on dialysis at the time of starting enteral tube feeds; this finding is similar to the poorer response to rhGH on dialysis [6]. It may be a feature of low power, with only 16 children in the dialysis subgroup, but it may also reflect a critical window of intervention during earlier stages of CKD where the most significant benefits in height can be obtained. It is also important to note that the dialysis subgroup had a higher median age; therefore, further study is required to elucidate whether the poor growth is due to dialysis or a higher age, or a combination of both.

Our study has some limitations; notably, its retrospective nature meant that there was a significant amount of missing data for biochemical parameters. Also, our power was reduced when using paired statistics, due to missing 2-year follow-up data in 10 children who had received a renal transplant and therefore data collection was stopped. In addition, the retrospective nature of the study means that data are not available on exact energy intakes before and after tube feeding, which would be useful information to have. The lack of a comparator group in this study limits our ability to draw firm conclusions for all of the outcomes assessed. A further limitation is the significant heterogeneity in the underlying primary renal diagnosis within our cohort. Whilst the inclusion of children with cystinosis may reduce the power of our study, as these children have poor growth that is often very difficult to manage [19], it was felt that their inclusion was relevant as they make up a substantial proportion of children with CKD and poor growth. Removal of the 7 children with cystinosis from the study cohort does not significantly alter the overall results.

Despite the above limitations, our study is the largest cohort to date assessing the impact of enteral tube feeding in older children with CKD. Enteral tube feeding is very common in children with CKD, but it is most often started before 2 years of age. Therefore, children starting enteral feeds after 2 years of age are still relatively uncommon (as a proportion of all children with CKD). A prospective study of this nature would therefore take a very long time and is unlikely to be feasible. Our study has also attempted to control for common confounding factors, such as rhGH use and renal transplantation by excluding these children from our analysis. We have also only studied pre-pubertal children, to avoid the varying age of entering puberty and its potential impact on Ht SDS.

In conclusion, enteral tube feeding commenced after 2 years of age in children with CKD is associated with improved Ht and Wt SDS, with stability of BMI during the second year. Younger children and those not on dialysis had the greatest benefit. We suggest that paediatric nephrologists and dietitians should consider enteral tube feeding in all children with CKD, particularly in children who have a lower than average BMI and children under the age of 6 years when significant improvements in height may be achieved.
